# COVID-19 Induced Brief Psychotic Disorder: A Case Report and Review of Literature

**DOI:** 10.1155/2022/9405630

**Published:** 2022-01-05

**Authors:** Sulaimon Bakre, Kritika Chugh, Oluwaseun Oke, Anita Kablinger

**Affiliations:** ^1^Virginia Tech Carilion School of Medicine, 2017 S Jefferson St, Roanoke, VA 24014, USA; ^2^Children's Health, 1935 Medical District Drive, Dallas, TX 75235, USA

## Abstract

The COVID-19 pandemic has significantly impacted people around the world, with asymptomatic infection to severe diseases and death. There is an increasing incidence of mental health problems in patients diagnosed with COVID-19. There are some studies that discuss possible mechanisms responsible for psychotic disorders due to coronavirus as well as risk factors for developing psychosis in patients infected with the virus. We report the case and a review of the literature in a 29-year-old female with no past psychiatric history who was diagnosed with a brief psychotic disorder following infection with COVID-19.

## 1. Introduction

Since identification of the SARS-Cov-2 virus in December 2019, COVID-19 infection has affected over 209 million people (World Health Organization), imposing a significant physical and mental health burden on communities around the world. Though many of the psychiatric symptoms patients experience may be due to social and psychological stressors related to the impact of the virus, there is evidence that the virus itself can trigger the onset of neuropsychiatric syndromes. This is not surprising information. Other strains of coronavirus, such as the one responsible for the 2003 severe acute respiratory syndrome-associated coronavirus (SARS-CoV) epidemic, have also been connected to acute psychotic disorders. Some of these patients at that time presented with acute psychosis with symptoms of agitation, persecutory delusions, and hyperactivity, which caused difficulty with infection control measures and cooperation with medical management. Studies indicated that this psychosis was not simply due to steroid toxicity, as a family history of psychiatric illness was a strong risk factor for SARS-related psychosis [1].

Here, we discuss a case of a patient with COVID-19-induced brief psychotic disorder with no prior psychiatric history.

## 2. Case

A 29-year-old Caucasian female with no past psychiatric history was initially admitted for sore throat, fever, headache, and loss of smell and taste and was diagnosed with COVID-19. She reported prolonged thoughts about taking her life and had attempted suicide by overdosing on 20 tablets of acetaminophen. She presented with disorganized thoughts, stating that she was unaware of her wife's pregnancy and bleeding. When asked how she knew that her wife was pregnant, she responded that her wife had pain in her vagina. The patient also believed that she herself was pregnant and felt her baby “could not breathe and had lost too much blood.” She later stated that she wanted to go home to see her husband. She could not explain how she had both a wife and a husband at the same time.

She mentioned that she had been feeling depressed for the past two weeks, had trouble sleeping, and had not slept in two days. She endorsed low energy, poor concentration, and poor appetite but denied current suicidal thoughts. She reported visual hallucinations of “black stuff” coming out of her mouth and auditory hallucinations of Satan telling her to “fight” and telling her to “stay” at the hospital against her will. The patient felt suspicious of the doctors and nurses as she suspected they were abusing illicit drugs. She stated that her father molested her and she has nightmares, flashbacks, hypervigilance, and depersonalization. She admitted to smoking cannabis for the past two weeks, a ball pack daily. She added that she abused a lot of drugs while she was younger, specifically cocaine, methamphetamine, and alcoholic drinks every now and then. Regarding family history, her biological father was diagnosed with bipolar disorder.

Collateral information acquired from the husband and patient's mother revealed otherwise. We found out that the patient was fine in the past few weeks prior to her admission until her husband got diagnosed with COVID-19. Two days prior to presentation, the patient woke him up in the middle of the night stating that “we need to go meet Jesus, you got evil in you.” She did not sleep for about two to three days but was able to sleep for about 5 hours the night before presenting at the hospital. Patient was worried that her husband was going to die. The husband stated that the patient never had suicidal ideations and did not overdose on acetaminophen. She took acetaminophen for headache the night prior to admission which was the usual dose. She was never molested by her father. She does not smoke marijuana. She was never depressed. She wants to get pregnant but has not been pregnant. She was never admitted into inpatient psychiatric unit even though she endorsed having four past psychiatric admissions. She had never seen a psychiatrist or had any psychotropic medication trials.

On mental status, she is a young female who was restless and in emotional distress. Speech was normal in volume, rate, and rhythm. She was oriented to place and person but not to year or month. Attention and concentration were normal. She was responding to internal stimuli and was talking to Satan who was telling her to fight. She had paranoid delusions towards the medical staff. As mentioned above, she had disorganized thoughts. She had good registration but scored 0/3 on delayed recall. Abstract thinking and judgment were good. Urine drug screen and pregnancy test were both negative. WBC was 9.8 K/*μ*L, acetaminophen level was <15 *μ*g/mL, and urinalysis was positive for leukocyte esterase and numerous squamous cells, but rare bacteria. Urine culture was positive for Escherichia coli, but the patient was asymptomatic. Her presentation was consistent with a brief psychotic disorder which was likely precipitated by COVID-19; however, delirium could also be a differential diagnosis.

The patient was started on olanzapine 10 mg daily for psychosis. She started to feel better and be more like herself, more organized, and behaviorally appropriate on the fourth day of admission. She denied significant mood symptoms, suicidal thoughts, and perceptual disturbances, and her husband also noted that she sounded more like herself over the phone. The patient was switched to aripiprazole 10 mg daily due to concerns for metabolic problems and increased weight gain which is a more common side effect of olanzapine. The patient was discharged home in stable condition on the fifth day.

## 3. Discussion

Studies have shown that coronavirus infections such as SARS and MERS, alongside with COVID-19, can lead to psychiatric symptoms in patients. A systematic review of 72 articles found occurrences of anxiety, depressed mood, insomnia, and impaired memory during the acute illness of the infections [2]. Persecutory delusions, severe paranoia, anxiety, decreased sleep, and suicidal thoughts have all been reported as presentations of psychosis in COVID-19 patients [3–5]. Here, we present a case of a 29-year-old female with no past psychiatric history that developed psychosis after developing COVID-19. With no past psychiatric history or administration of psychosis causing medication, such as corticosteroids, to our patient, it suggests that the COVID-19 virus may have been responsible for triggering the psychosis seen in our patient.

One possible mechanism for this could be due to the effect coronaviruses have on the nervous system. Coronaviruses are regarded as neurotropic and can enter the brain, therefore triggering an inflammatory response [6], resulting in release of inflammatory cytokines like IL-6, TNF-*α*, and IL-1*β* that have been found in patients with first episode psychosis [7], which may be the reason for the psychosis seen here. The virus is thought to enter the central nervous system (CNS) via the hematogenous route or peripheral neurons, relying on retrograde axonal transport after which it binds to the angiotensin-converting enzyme 2 (ACE2), the cell entry receptor on both neurons and glia [8]. Though the overall levels of this receptor within the CNS are low compared to other organs (for example, the lungs), it is still implicated within the pathogenesis for the neuropsychiatric symptoms in patients with coronavirus [8]. Within the CNS, the overall expression of ACE2 receptors is relatively high in dopaminergic and serotonergic nuclei, glutamatergic neurons, lateral ventricles, and the substantia nigra, which are areas that are implicated in schizophrenia [8]. One possible hypothesis is that these areas, due to their relatively high concentration of the ACE2 receptors, are more vulnerable to the virus, which may shape the neuropsychiatric symptoms we see in COVID-19 patients [8].

Another factor to consider is the psychiatric family history of the patient, as the patient had a father with bipolar disorder. A family history of psychiatric illnesses has been implicated in SARS-related psychosis as mentioned above.

As with most new-onset psychotic episodes due to COVID-19, the treatment of choice involves the use of antipsychotic medication [3–5]. Once we determined our patient was having a psychotic episode, we proceeded to treat her with olanzapine. She required no other treatment at this time.

## 4. Conclusion

There has been several literature discussing the neuropsychiatric presentations of coronavirus infections; however, only a few have been specifically geared towards the COVID-19 virus. There is a possible association between COVID-19 infection and brief psychotic disorder in patients with no past psychiatric history; however, further association studies need to be done to assess a relationship. This case report will contribute to the knowledge base as we continue to understand the neuropsychiatric presentations of the COVID-19 virus.
